# Mr. Hey, Jun. on Puerperal Fever

**Published:** 1824-09-01

**Authors:** 


*A Treatise on the Puerperal Fever, illustrated by Cases, which occurred in Leeds and its Vicinity, in the Years* 1809—1812. By WILLIAM HEY, Jun. Member of the Royal College of Surgeons in London, and Surgeon of the General Infirmary, and of the House of Recovery, at Leeds. pp. 238.

IN spite of the attention which has been paid to the subject of puerperal diseases within the last few years, we are persuaded that there is no department of physic so defective at this very moment, or which so imperatively demands a renewed investigation. The subject must be again taken up; the symptoms must be accurately noticed, and accurately compared with the effects of remedies, and with the morbid appearances in fatal cases, before the confusion with respect to the diagnosis, and the difference of opinion with respect to the mode of treatment, which have so long prevailed, and which still prevail, especially in regard to what has been called "the puerperal fever," can be removed. For want of such an inquiry as this, cases, totally different in their nature, have been considered and treated, and even published as the same. We do not think the excellent Treatise of Mr. Hey even, quite free from this charge, although few medical works have appeared to us to display so much candour, and so much of the real spirit of inquiry and love of truth.

In all the late publications on puerperal fever, the cases treated have been too hastily and indiscriminately considered to be *the same;* and very frequently their authors do not appear to have imagined, that a difference of opinion respecting their nature could exist in the breasts of their readers, and have therefore, scarcely thought it necessary to enter into that accurate and minute detail of symptoms which alone could remove such doubts, should they exist. This charge we fear is also, in some degree, imputable to some of the cases treated and given by Mr. Hey.

The profession is still in want of a treatise, the principal object of which should be to trace these distinctions in the nature and characteristics of the various and different puerperal diseases; we still want a treatise on these diseases conducted on the basis of *morbid anatomy*, in conjunction with the principle just alluded to. Perhaps the chief defects of Mr. Hey's work have arisen from the total absence of *post mortem* examinations.

Notwithstanding the observations which we have thus made, and which we may hereafter make in our *analysis of the cases* contained in Mr. Hey's work,—our main object in this review,

—we still regard this gentleman's treatise as forming a most valuable addition to our stock of information on the subject of puerperal diseases; and we think no one can rise from its perusal without feelings of the utmost esteem for its author.

Before we proceed to the immediate object of our review, as just stated, we propose to make such preliminary observations on the differences *existing* in the nature of puerperal cases, as may, like so many rallying points, assist us in our progress, and our readers in apprehending the scope of our arguments. We conceive then that the most common of all puerperal cases, are those which arise out of the state of the bowels; these cases have been slightly noticed by Mr. Burns and Dr. Granville, as intestinal fever,—and by Dr. Marshall Hall, in an express treatise on the subject, as intestinal irritation; the affection is constantly mistaken for peritonitis. The second class of puerperal cases, in the order of their frequency, is that of inflammations,—and they may be divided into the more diffuse inflammation of the peritoneum, and the more confined inflammation of the *substance* of the uterus and its appendages, and of their peritoneal covering. In the third place, we have very frequently to observe the effects of loss of blood, either induced by uterine hæmorrhage or from the use of blood-letting,—a subject scarcely noticed in any treatise except the little brochure by Dr. Hall, already mentioned. In the fourth place, we are constrained to draw the attention of the profession to the numerous examples of *mixed cases*, a subject, to the practitioner, of the utmost importance,—from their constant occurrence in practice, from their often puzzling character, and from the total silence on such cases in all systematic treatises. Lastly, we come to the subject of epidemic puerperal fever,—and it would be well, we think, for many mothers, if we had sufficient influence with the profession, to abolish the name entirely from our books, substituting the term epidemic puerperal peritonitis for one well-ascertained form of it—and any other appropriate term which a careful investigation of the subject might render necessary for other forms;—for we still consider this matter as *sub judice*, and would hesitate to determine whether *every* case of *epidemic* puerperal disease, be in fact inflammation of the peritoneum; and we would, in an especial manner, remind our readers, that during an epidemic puerperal peritonitis, cases of a sporadic and different nature may and do occur.

There is still another view to be taken of puerperal diseases. For they sometimes affect the *head* entirely, sometimes the *abdomen*. What is the separate nature of these affections? It has been usual to speak of puerperal *phrenitis;* and doubtless such a disease may occur, just as pleuritis may occur, in the puerperal state; but we are satisfied that it is a far more rare puerperal disease than has been formerly, at least, supposed. All the symptoms generally considered to characterize phrenitis, occur from intestinal irritation, and some of them from loss of blood. There is, besides that peculiar disease—puerperal delirium. All these cases must be distinguished from phrenitis. On the subject of abdominal affections we need not say more than we have already done, our present object being not to treat fully of these affections, but merely to throw out a few hints for the better understanding of our subsequent remarks. For the same reason, we omit for the present any thing more than a mere allusion to those diseases—chiefly convulsions and apoplexy—arising from the pressure of the gravid uterus and of a loaded intestine, before delivery, and from the efforts of delivery itself—and to some other affections occurring in the puerperal state, but obviously distinct from the subject of the treatise before us, to which we must now return.

The Leeds epidemic is supposed to have spread itself, with some intermissions, over the years beginning with December 1809, and ending with the latter end of 1812; Mr. Hey has, however, added in an appendix several cases of puerperal disease which occurred in 1814. Some of Mr. Hey's cases are altogether defective in regard to the symptoms;—others are full and ample and enable his readers to judge for themselves, in some degree, of their nature; with these we shall now occupy ourselves and earnestly request the attention of our readers to a variety of remarks to which they have given rise.

We transcribe Mr. Hey's first case.

"The first case which occurred in my practice, was that of a young married lady, who resided in an open and healthy situation at a little distance from the town. She was safely delivered of her first child, after an easy and natural labour, in the evening of the 9th of December, 1809; and remained quite well till the afternoon of the 11th, when she was seized with a rigor. I visited her soon afterwards, and found her in a state of perspiration—pulse at 120, and not full. She had so little pain in the abdomen, that, had I not been minute in my inquiries, she would not have noticed it; pressure on the hypogastric region did not excite much uneasiness.

"Though the slight degree of pain in this case might have tended, under other circumstances, to mislead my judgment, I was not unsuspicious of the nature of the disease; my attention to it being particularly excited by the recent death, in child-bed, of two ladies in the suburbs of Leeds.

"My father had long been in the habit of treating cases of puerperal fever in a manner somewhat similar to that which Dr. Denman recommends. After freely evacuating the bowels, and occasionally drawing blood from the arm, he prescribed such a dose of some saline purgative, to be taken every morning, as might procure four or five stools in the course of the day; and endeavoured, in order to recruit the strength of the patient, to gain a respite at night by administering an opiate.

"By regularly pursuing this plan, I have seen some bad cases of puerperal fever cured, in which the pulse was at one hundred and forty, or upwards, and the abdomen considerably enlarged. The same treatment has been alike successful, when, in the course of the disease, a spontaneous diarrhœa has arisen; with this difference only, that a smaller dose of purgative was usually sufficient, in proportion to the length of time and degree, in which the diarrhœa had subsisted. Though sometimes the diarrhœa, being in a great measure the effect of irritation, was rather moderated than increased by a proper dose of some mild purgative.

"I saw nothing in the case under consideration to forbid a similar treatment. Venesection did not appear to be indicated; but recourse was had to cooling purgatives, salines, and opiates, as the peculiar circumstances of the case seemed to require.

"The treatment was, in the first instance, attended with all the success that I could wish; the uneasiness in the abdomen was removed, the pulse came down between thirty and forty strokes in a minute, and I had the best hopes of my patient's recovery.

"But I had not yet learnt the intractable nature of an *epidemic* puerperal fever; for this remission, in accordance with a remark of Dr. Gordon, proved 'only a respite, during which, the disease is preparing strength to return again, in order to renew the conflict with redoubled vigour, when it will not be in the power of art to check its impetuosity.'*

"After a few days, the pain, which before had been trifling, returned with greater severity, and the abdomen became sore and tumefied. Purgatives and opiates were again employed, and the former with evident and repeated advantage.

"My father frequently saw this patient with me; a physician was also requested to assist us with his advice; and, before the termination of the disorder, a second physician was consulted; but all was in vain. Notwithstanding the various checks which the complaint received, every remission was less complete than the preceding, and every fresh attack more severe. The pain and swelling of the abdomen increased; an obstinate diarrhœa came on, in which the stools were sometimes dysenteric, sometimes feculent, but watery, and generally accompanying the paroxysms of pain, which for a time was always diminished by them. The lochia were sometimes nearly suppressed; at others, they appeared afresh. The faculties continued clear, and the tongue moist, till within a short time of the fatal termination of the disease, which happened on the tenth day.

"Next to the obstinacy of the disorder, nothing was so remarkable in

* "Treatise on the Puerperal Fever, p. 86."

this case, as the relief procured by purgatives, which was such as to give us the hope, more than once of a favourable issue; and the use of them, I doubt not, prolonged the life of the patient many days. For, whenever, through fear of the strength being exhausted, or from an idea that the diarrhœa constituted a part of the disorder, any attempt was made to restrain it, an increase of the pain invariably followed; on the contrary, when the purgatives were repeated, some abatement of pain was the consequence, sometimes even before they operated. Cinchona and cordials were prescribed towards the close of the disease; but without any advantage." 40.

We think there can be little doubt but that this case was one of *peritonitis;* and we are not surprised that without blood-letting any treatment should be unavailing; the treatment adopted is applicable to another form of puerperal disease. It is observable that the *head* was not affected; and we notice this particularly, because we believe such affection frequently denotes a case different from simple peritonitis, in which latter disease it is plainly not *essential.*

The fourth and fifth cases are also evidently inflammation. The sixth is given by Mr. Hey as a fair example of the disease, and we therefore copy it. It is to be remarked, that the tongue, skin, and head are not affected.

"Mrs. W—— was brought to bed of her eighth child on the 26th of January 1810, at midnight. Her labour was natural, and rather quick; and was attended with a moderate and proper discharge. She was affected with after-pains for a few hours after delivery, which then left her, and she remained easy.

"Being desirous to avoid whatever might prove an occasion of irritation to the abdominal viscera, or cause a determination of blood to them, we purposely abstained, in this case, from prescribing any purgative medicine. At five o'clock, *p. m.* on the twenty-eighth, she was found to continue perfectly well. Her pulse was then at 72.

"29th. I was called to visit her at eight o'clock in the morning, and was at the same time informed, that she had passed a very bad night. After suckling her child for some time, she had been seized about one o'clock (forty-nine hours after delivery) with a shivering fit, accompanied with severe pain in the abdomen; and to the circumstance of giving suck *she* attributed her disorder. The pain had returned with great severity at short intervals throughout the night, leaving, during its remissions, extreme soreness in the abdomen. I found the pulse at 120, the tongue clean, and the abdomen very tender, but not enlarged. The skin was cool, and the face pallid. She complained of thirst. About six o'clock she had experienced some degree of nausea. I ordered a purging clyster to be injected immediately, and a table spoonful of ol. ricini to be taken every two hours, no stool having been procured since the delivery.

"Anxious to afford every assistance to my patients, and unwilling that the whole responsibility should rest upon myself, in these truly alarming cases, I immediately requested a consultation. The physician who was first sent for, being from home, another was called in; but some delay was necessarily incurred by this circumstance.

"*At half-past* 2, *p. m.* I found the patient somewhat easier, and the pulse reduced to 110, though the oil had not yet operated. She had taken three doses of it. The clyster had not been well managed; but the little that had passed, had brought away some hardened fæces.—Another was injected.

"*Five, p. m.* A fourth dose of the oil had been taken at three o'clock; but no stool had been procured, and the last injection was still retained. Some flatus had been expelled *per anum.* An enlargement of the abdomen was now evident, but at what period it commenced I cannot exactly say.

"The physician who saw the patient in the morning, and now met me in consultation, had just witnessed with me the rapid progress of the disease, as related in the preceding case; and, as that was thought to have a stronger analogy to the puerperal fever described by authors as a species of low fever, than to a case of phlegmonous inflammation, it was judged proper to prescribe accordingly.

"The purgative already taken was relied upon for evacuating the bowels, as they had not hitherto been found difficult to be acted upon in this fever, if purgatives were given at the commencement of the disease; and the following medicines were prescribed.

℞. Pulv. cinchon. ʒss. 2dâ quâque horâ sumend. cum coch. iij julep. infra præscr.

℞. Mistur. camphoræ ℥vj.

Liq. ammon. acet. ℥ ij. M.

"I had indeed found nothing so beneficial as purging; but I could say nothing of my success; and therefore could not object to the trial of other and different means.

"*At* 8, *p. m.* the physician who had first been sent for, accompanied us to visit the patient. The swelling of the abdomen had increased, and the pulse was at 120. As but little opportunity had been afforded for the trial of the medicines so lately prescribed, it was agreed that they should be continued. An anodyne fotus, composed of twelve ounces of a decoction of poppy-heads and four of spt. camphoræ, was directed to be applied to the abdomen.

"30th. I visited the patient at seven in the morning. She had passed another very bad night. Since twelve o'clock the pain had become much more severe. She had vomited the medicine at four o'clock, in consequence of which it had not been repeated. The tongue was dry. As no evacuation by stool had been procured, another clyster was injected; and, in pursuance of the plan of treatment which had been adopted, I only ordered a draught with decoct. cinchonæ and thirty drops of the tincture, till I should meet the physicians.

"*At half-past* 9, *a. m.* we saw her together, and found her in no respect relieved. The vomiting continued, and the pulse was at the rate of 130. A saline mixture was directed to be given in a state of efferves-cence.

" *Two, p. m.* Every thing had been rejected almost as soon as taken. The pain of the abdomen had increased, though its distention was not greater, and the cries of the patient were very distressing. Opiates were given both in a liquid and solid form, but without any advantage.

" *Eight, p. m.* She was becoming delirious, her pulse was not to be felt, and she died the same evening. I do not know the exact time of her death, but it must, I think, have been within forty-eight hours from the attack of the disease." 62.

In the third case there were heat of the surface, and a white tongue, and the head was affected, after a violent rigor, symptoms which frequently concur in the same case, and which have appeared to denote the addition of intestinal irritation to the state of inflammation.

In the seventh case, inflammation seems to have begun and to have subsisted for three months before delivery, in spite of laxatives, opiates and bleeding. It is to be regretted, that Mr. Hey has not specified in what measure these remedies were employed; and it is almost surprising that active antiphlogistic means were not resorted to in this case, at least, both before and after delivery. The case is extremely interesting in every point of view, and we cannot do better, we think, than transfer it to our pages.

" Dr. Denman observes that there are instances of puerperal fever being formed before delivery. I have mentioned one such instance,* in which the patient was attacked with the symptoms of it a few days before labour, and died within twenty-four hours after her delivery. I have also alluded to the following case, in which there appeared a strong predisposition to the disease during the latter part of pregnancy.†

" For nearly three months before her confinement, but more especially during the last five or six weeks, Mrs. K—— suffered much from very unusual pains in the abdomen. She was seldom quite easy; and every day she had one or two paroxysms of severe pain, which continued several hours. They came on at different and uncertain times, and affected chiefly the hypogastric region, but sometimes the epigastric. They were often alleviated by rest in a horizontal posture, though not unfrequently they came on in bed; but when the pain was most subdued, it left the abdomen very sore. Laxatives, opiates, and bleeding, were the principal means tried for its relief, but with little success.

" February 5th, 1810, in the evening, she was delivered of her first child, after a lingering, though not a severe labour, of three days. During the two first, the pains were slight, but distressing in consequence of

* See p. 26. † Ibidem.

the soreness of the abdomen. On the third day, the labour was more natural, and less distressing. I frequently visited her, but only remained by her during the last two hours. On the morning of this day, the pulse was at 72; after delivery, at 100.

"6th. She had passed the night without much sleep, having had frequent pains resembling after-pains, and, in the intervals, great soreness of the abdomen. The uterus was easily distinguished reaching nearly to the navel, and shewed great tenderness when touched. She had much difficulty in turning herself in bed. She complained of thirst, but had not much heat. The tongue was rather white and furred. Pulse at 80. I forbad every thing heating in diet, and ordered a draught to be taken immediately, containing rhubarb and tartarized soda, of each a dram.

"*Half-past* 2, *p. m.* The pain and soreness of the abdomen had rather increased, and the draught not having operated, a mild clyster was injected. Pulse at 98. I prescribed a saline draught with vin. antimon. gutt. x, to be taken every two hours.

"*Half-past* 6, *p. m.* A very copious stool, containing much solid fæces, had been procured, and the pain was greatly diminished. Pulse at 108.

"*Ten, p. m.* The pain had become more severe again, shooting into the hips, thighs, and even to the toes; the soreness of the abdomen, and the difficulty of moving had also increased. As she had had but one small additional evacuation, I ordered another draught with magnes. sulphas and manna, āā ℥ss, to be taken immediately; and the saline draughts to be afterwards continued with the addition of ten grains of pulv. ipecac. comp. to each of the two first.

"7th. She had got no sleep in the night, except half an hour since six o'clock. Two pretty copious stools which she had in the night, were of a dysenteric kind, and contained no fæces. The fur of the tongue was partially cast off in the middle. The state of the abdomen remained throughout the day much the same as before. The intention of endeavouring to excite and keep up a gentle diarrhœa, was still pursued; and the opening medicines were varied in their kind and dose, as the symptoms seemed to indicate. About noon a vomiting came on, which was removed before night; and, good evacuations by stool being also procured, the patient felt much relieved. A fresh discharge of lochia took place during the day. The pulse was at 130 both morning and evening. A saline draught with thirty drops of tinct. opii, was ordered to be taken at bed-time; and a saline mixture, at intervals, in a state of effervescence.

"8th. She had passed a pretty comfortable night, and had slept a good deal. Pulse at 112. The purgatives to be continued.

"*Four, p. m.* Four or five small natural stools had been procured; and the pain, swelling, and soreness of the abdomen, had much abated. The tongue was clean in the middle. An habitual cough, which seemed, from the period of labour, to aggravate all the other symptoms, still continued very troublesome, and was attended with a large expectoration of frothy phlegm. Pulse at 120.

"I was sent for between seven and eight o'clock in the evening in consequence of a fresh attack of vomiting. She complained of soreness and a sense of fulness in the pudendum, which induced me to examine the parts; when I found a patch of erysipelatous inflammation on each of the *nates*, and an œdematous enlargement of the *labia pudendi.* A fetid ichor was discharged from the vagina. The urine was generally forced away by the cough, which might tend to increase the inflammation. The following medicines were prescribed.

℞. Decoct. cinchon. ℥iss.
Ammon. carbon. gr. v.
Tinct. opii. gutt. x. M. fiat
haustus statim sumendus, et, horis duabus elapsis, repetendus cum tinct. opii gutt. v.

"*Half past ten, p. m.* The first draught had been taken, and the vomiting had ceased. The pulse was at 134; but it was probably quicker in consequence of the patient having just been moved. The draughts were ordered to be repeated every two hours, with three drops of tinct. opii in each; and a table spoonful of wine to be given now and then. A cooling ointment was prescribed for the inflammation.

"9th. She had slept some hours in the night, and all the symptoms were relieved. Pulse at 106. Tongue cleaner and more moist.

"*Evening.* In the afternoon the cough had become more troublesome, and was accompanied with a darting pain in the abdomen, the swelling and hardness of which had increased. The vomiting also had returned. An opening draught had procured two natural loose stools, and the vomiting was relieved, but the pain continued the same. The erysipelas had become more extensive, and the patient was hot and restless. Pulse at 120. Two grains of opium were ordered to be given with an interval of four hours, and the draughts to be continued, with the addition of a tea-spoonful of lemon-juice.

"10th. A considerable remission of the symptoms had again taken place in the night. The skin had become cool, and the tongue cleaner. The pulse was soft, and beat no more than an hundred strokes in a minute. This truce, however, was not of long duration; the pain and vomiting soon returned, the distention of the abdomen increased, and before night the pulse got up again to 120.

"From this time the disease made a regular progress without any material remission. Cordials, anti-emetics and opiates, were administered with little effect. The erysipelas continued to spread, and the vomiting, pain, and distention of the abdomen, grew worse and worse; till, on the evening of the twelfth, just seven days from the delivery, death put a final period to them. The tongue had become quite clean, and, if the patient was at all delirious, it was not until very near the fatal close of the disease.

"We are informed that, in the Puerperal Fever of Aberdeen, 'a very frequent crisis of the disease was by an external erysipelas;' and that 'one of the most favourable symptoms is an erysipelas on the extremities, or abscesses on different parts of the body; for such are certain signs of a salutary crisis.' I never met with an instance of either critical erysipelas or abscess, in the Epidemic at Leeds; nor do I recollect any case, except the foregoing, in which erysipelas appeared at all. In this it was unfortunately not critical." 69.

The eighth case is full of interest, and we copy it both on that very account and for the sake of the observations which we have to make in regard to it.

"Mrs. N——————, residing at a solitary house in the country, about three miles from Leeds, was brought to bed in the night of the 7th of February 1810, after a short and easy labour. She was a middle aged woman, and had borne many children. On the ninth, I gave her a gentle laxative, which had the desired effect. On the morning of the tenth, I found her sitting up to suckle her child; she seemed unusually well, and so she remained till the end of six days.

"14th. I was called up at one o'clock in the morning to visit her, and was informed that, having gone to bed quite well, she was seized at eleven *p. m.* with a shivering fit, which was succeeded by a great degree of heat, and pain in her body (shooting also into her hips and thighs) resembling labour-pain, but continuing without any perfect intermission. She complained also of much pain and throbbing in her head. Though the heat had begun to abate before my arrival, the skin was still hot and dry; but soon afterwards a profuse perspiration succeeded. The tongue was furred and very white; and the pulse beat at the rate of 150. The breasts were flaccid, and I desired that the child might not be allowed to suck. The abdomen did not shew any tenderness upon pressure. The lochia had returned afresh on the preceding morning, and in the evening she had had a natural and easy stool.

"The want of success which had hitherto attended the treatment of the disease, induced me immediately (though it was night) to consult with my father on the management of this case. We were satisfied that no remedy had done so much good as purging, yet it had not proved sufficient for the cure of the disease. We therefore thought it proper to add such means, as might tend to allay the local irritation, without much interfering with the operation of purgatives. With this intention, we ordered a draught with rhubarb and tartarized soda, of each a dram, to be taken immediately; a small clyster with forty drops of tinct. opii to be injected; a large blister to be applied to the abdomen; and a saline draught to be taken every two hours.

"*Half past two, p. m.* The pain had somewhat abated before the medicines arrived. After the injection of the opiate, it had gone off entirely, and had not returned. A slight vomiting had come on after taking the purging draught, and probably a part of it had been rejected. A degree of chilliness succeeded by heat, had returned about one *p. m.* Pulse at 126. I prescribed the following mixture;

℞. Sod. tartariz.—mannæ, āā ℥ss.

Tinct. senn. ʒij.—Aq. fervent. ℥ij.

Sumat tertiam partem alternis horis; and ordered a domestic clyster to be injected. I took off the blister, which by mistake had been applied to the back.

" *Nine, p. m.* Two doses of the mixture had been taken, and had procured three loose feculent stools. A degree of nausea had once been felt after taking some broth. Pulse at 134.

" 15th. *Half-past one p. m.* The patient had passed a very comfortable night, and had slept a good deal. She remained free from pain and soreness in the abdomen; and the secretion of milk seemed to be returning in the breasts. The tongue was cleaner. Pulse at 104. She had had one copious stool of solid fæces in the night, but none since that time. The saline draughts were ordered to be taken every four hours, and the purging mixture in such doses as to keep open the bowels; also a clyster to be injected in the evening. A table spoonful of wine in gruel was allowed to be given now and then.

" 16th. The injection had produced two plentiful stools containing large lumps of solid fæces. The patient complained of more pain in her head, and her tongue was furred. Pulse at 96. The medicines were ordered to be continued; another clyster to be injected in the evening; and the feet to be immersed in warm water.

" 17th. *Four, p. m.* Notwithstanding a pretty good night, she had not been so well this morning. The pain in her head continued; and she had several times experienced an acute shooting pain in the region of the uterus, which did not remain, but had produced some degree of soreness in the abdomen. She complained of thirst; the tongue was a good deal more furred, and the pulse at 104. Several loose evacuations had taken place in the preceding evening, but none after nine o'clock.

" Ordered, the opening draught to be given immediately; and the clyster in the evening, if the draught should not operate before nine o'clock. The patient having taken a dislike to the saline draughts, the carbonate of potass with lemon-juice, to be taken in a state of effervescence, was substituted in their place.

" 18th. The opening draught and injection had failed to operate. The abdomen was distended and hard, but not painful. Some degree of nausea had come on in the night, but had not produced vomiting. The skin was cool and pallid. The tongue was covered with a brown fur, and the pulse was at 112. A repetition of the clyster and opening medicine was directed.

" *Six, p. m.* A copious stool had been obtained, containing a good deal of mucus; and much flatus had been expelled *per anum.* The abdomen was soft, easy, and considerably reduced in size. Countenance good. Pulse 114.

" 19th. The patient had passed a very good night, and was in all respects better. The pain in the head and abdomen, and the enlargement of the latter, were quite gone. The fur of the tongue was coming off, and the pulse was at 98. A clyster had been injected, and had procured a proper evacuation.

" About noon, she was seized with a cold fit, scarcely proceeding to a rigor, which was succeded by great heat, a very frequent pulse, and pain in the head. A second clyster was injected, which operated and gave sensible relief. I ordered an opening draught to be taken in the evening, and the clyster to be repeated if necessary.

"20th. The draught and injection had both been given, and an evacuation procured by each, containing lumps of hardened fæces, which had the appearance of having remained in the bowels for some time, and had probably been the cause of the cold fit. The head was quite relieved; the fur was cast off from the tongue; and the pulse was reduced to 90. As there was some appearance of languor, a table spoonful of wine was directed to be taken frequently in some nourishing liquid.

"21st. No complaint, except soreness of the tongue and fauces, which were affected with aphthæ.

"On the 22nd, the patient, having been rather longer than usual without a stool, was again attacked with chilliness succeeded by heat, but in a much less degree than before. She was relieved by an injection; but this attack occasioned her a restless night.

"From this period, she recovered without any relapse; but was some time in regaining her usual strength, on which account she took various tonic medicines." 76.

It is to be remarked, that on the first day there were, in this case, rigor, great heat and dryness of the surface, much fur and whiteness of the tongue, and much pain and throbbing of the head, whilst the abdomen was free from tenderness on pressure; and again "chilliness succeeded by heat." On the next day, the abdomen "remained free from pain," and solid fæces had been passed. On the third day, "large lumps of solid fæces" were passed, and there was "more pain of the head," but still no mention of pain in the abdomen. On the fourth, an acute shooting pain in the region of the uterus is first noticed; on the fifth the "abdomen was distended and hard but not painful;" and in the evening, "soft, easy, and considerably reduced in size." On the sixth day the pains and enlargement of the abdomen were quite gone; in the evening there were rigor, great heat, frequent pulse, and pain of the head,—greatly relieved by a glyster; and on the succeeding day the patient passed "lumps of hardened fæces, which had the appearance of having remained in the bowels for some time."

We have been thus minute in our account of this case, because we are quite certain that it was *not* a case of puerperal fever or peritonitis. There is scarcely a symptom of abdominal inflammation; but there is every symptom and every kind of evidence of intestinal irritation and its effects, as described by Dr. Hall. The patient recovered, too, without the use of the lancet. All the previous patients had died under the same neglect of this all powerful and all essential remedy of inflammation. In the present case, too the attack and the symptoms such as they were, were severe; and we think that had they been inflammatory, nothing but the lancet could have saved the patient. But it is our office to give hints only, and we recommend and leave the case to the earnest consideration of our readers.

Not less instructive is case the ninth.

"June 18th 1810, I was sent for to Mrs. B——— a stout middle aged woman, living at a little distance from the town, who had borne several children, and was then in labour. The early part of the labour proceeded quickly, but the pains declining in strength, the latter part was slow. The placenta separated spontaneously, and was expelled by the natural efforts; but the uterus did not contract well afterwards, which occasioned too great an effusion of blood. However, by keeping up a compression with the hand on the fundus uteri for about an hour, the hæmorrhage was considerably restrained, and I left my patient apparently doing well.

"In about an hour, I received an urgent call in consequence of a fainting; and found the uterus much distended with blood. I removed the coagula from the vagina; and, by gently stimulating the os uteri with two fingers of one hand, and compressing the fundus with the other, a good contraction was produced, and the hæmorrhage ceased. The patient remained languid, but had no more fainting. Pulse 120.

"19th. No complaint but languor arising from the loss of blood. Pulse the same.

"20th. The strength had improved, but the pulse had rather increased in frequency. Ordered a gentle laxative.

"21st. *Eleven, a. m.* The laxative had procured three good evacuations, two of which were loose. The pulse had come down to ninety-six, and was full and strong. I observed the tongue to be dry in the middle.

"*Three, p. m.* Not long after my visit in the morning, the patient had been affected with a slight chilliness, which was succeeded by heat, vomiting, and a continued, though not violent, pain in the abdomen. She complained of soreness when the abdomen was touched; and the uterus, somewhat enlarged, was distinctly to be felt above the pubes. The skin had now become cool. I directed a purging clyster to be injected immediately, and a saline mixture to be taken every two hours in a state of effervescence.

"At this time I had not seen Dr. Gordon's Treatise on the Puerperal Fever of Aberdeen; for it was not much known in Leeds. But I had read the short account of it contained in Thomas's Modern Practice of Physic; and the last case which had occurred to me, having exhibited evident marks of acute inflammation, I was strongly inclined to make trial of bleeding. This inclination was strengthened by reflecting on the small success which had hitherto attended all other means; and still more so by the consideration, that purging, the other principal remedy of Dr. Gordon, was the only one from which I had seen clear and decided advantage. Unfortunately the present case was not favourable to the trial, the patient's strength having been previously reduced by a profuse hæmorrhage. No time, however, was to be lost; I determined therefore to repeat my visit soon, and to be guided by circumstances.

*Five, p. m.* The clyster had been given an hour and was still retained. The vomiting had not returned. The pulse was at 112; and as it was by no means a weak pulse, I determined to take a small quantity of blood from the arm, and to observe its effect. I took away seven ounces, and also applied a large blister to the abdomen.

" *At Eight, p. m.* My father visited the patient with me. She had parted with an astonishing quantity of fæces mixed with mucus. The pain came on at intervals, like after-pains; and was very moderate in the remissions, when she lay quite still upon her back; but the least motion of the body occasioned great uneasiness. The blood exhibited a very thick inflammatory crust, and the crassamentum was remarkably firm. The pulse was at 130, and hard. Under these circumstances, it was judged proper to repeat the bleeding to the same quantity.

" *Ten, p. m.* The second quantity of blood was not covered with so thick a crust, but the crassamentum was still more firm than the former. It was like a piece of liver; I could scarcely pierce it with my finger. The pulse had come down to 120, and was more full. She was lying upon her side, which she had not been able to do before, and was quite easy when at rest. She had complained all the day of great thirst. The tongue was clean, but still dry in the middle. A saline draught was ordered to be taken every three hours, and, as she had had several more loose stools, thirty drops of tinct. opii were added to the first.

" 22d. Throughout this day the pains were slight and distant, and their remissions almost complete, so that the patient could bear to take her nourishment sitting up in bed. The tongue was moist and clean. Some opening medicine being necessary, a dose of rhubarb and calomel was given, and the clyster repeated. By their joint operation a surprizing quantity of fæces was again discharged in the evening. The pulse was below an hundred in the morning, and in the evening at 116. As she had perspired a good deal, and appeared languid, the saline draughts were directed to be made with an ounce of decoct. cinchonæ. The anodyne was repeated.

" 23d. She had passed the night without any pain, notwithstanding which she had slept but little. Pulse at 110, and very strong. No more stools: clyster repeated.

" Having augured favourably of this case from the gradual and complete cessation of pain, it was with no less surprize than regret, that, in the evening, I found an intire new train of symptoms. The patient having been affected throughout the day with an irresistible propensity to sleep, from which she got no refreshment, awoke in the evening with pain in her head, accompanied with giddiness and ringing in the ears. Her face was flushed: her pulse at 132 and strong. She had had three loose stools, and had parted with a large quantity of urine. Some leeches were ordered to be applied to the temples; but finding, on a second visit, they had not been procured, I took three ounces of blood from the temporal artery. The saline draughts were directed to be made without decoct. cinchonæ, and a blister to be applied to the nape of the neck. Just before the bleeding the pulse was at 120, after it at 112.

"24th. I found the patient sitting up in bed to take some refreshment. She had slept several hours in the night. Her countenance was good. It was rather singular, that the left side of the head, from which the blood had been taken, was easy, but the opposite side painful. The crassamentum, as before, was extremely firm. Pulse 126. I took three ounces of blood from the temporal artery of the right side, and the evacuation greatly diminished the pain.

"In the evening she experienced a seizure somewhat similar to that of the preceding day. Having been visited by several friends, who had inconsiderately talked and read a good deal to her, she was suddenly affected with a sense of great confusion and noise in the head, accompanied with much heat and flushing of the face. Pulse 140. In consequence of the relief before experienced, she was very desirous to lose some more blood from the temples, and therefore, though the pulse appeared less strong, I took an ounce and a half from the temporal artery.

"The case having become more alarming by this relapse, a consultation was requested; and a physician who had attended several of these melancholy cases with me, was called in; my father also visited the patient with us. The pulse had come down to 120, and was evidently fuller since the bleeding. The crassamentum was as firm as before. It was agreed that the saline draughts should be continued, that a blister should be applied to the head, and the temples and forehead be frequently bathed with cold vinegar and water.

"25th. *Eight, a. m.* She had had no sleep in the night, but her head was rather more composed, and she was free from heat. Pulse 116. Some indications of a paralytic affection were now apparent. She faltered in her speech, and her tongue when put out, was drawn to one side. At noon the pulse got up to 140, she took little notice, and, though she sometimes spoke coherently, an answer to any question could scarcely be obtained from her; her mind also appeared much agitated.

"*At four, p. m.* the physician met us: it was agreed that a little wine whey should be given frequently, and the following medicine was prescribed;

℞. Spt. æther. comp. gutt. xxx.
Spt. ammon. comp. gutt. x.
Aq. puræ ℥iss. M.
fiat haustus tertiâ quâque horâ sumendus.

A draught with fifteen drops of tinct. opii was also directed to be taken at bed-time.

"26th. The night had again been passed almost without sleep; but the head was free from pain, confusion, and the sense of ringing. Pulse 116.

" *Two, p. m.* After three hours comfortable sleep, the head was not so well. The bowels were open, and the stools natural. Pulse 120.

" 27th. I was not able to see the patient myself on this day, and I neglected to minute any account of its occurrences.

" 28th. She had had no sleep in the night, and was very restless, with some degree of delirium. We found her incessantly talking, but could procure no answer from her to any question that was proposed. She refused all medicine. Pulse 120.

" In the course of the day the abdomen became tumid from flatus confined in the bowels; the tumefaction was unattended by pain or soreness, and intirely subsided as soon as evacuations were procured by an injection.

" *Ten, p. m.* She was in all respects worse. Her urine came away involuntarily; she had some rattling in her breathing, and appeared to be sinking. Pulse 132. Thirty drops of spt. æther. sulph. were ordered to be given now and then as a grateful cordial.

" 29th. We were agreeably surprized to find our patient much better. During the night she had been able to retain her urine, and had made a large quantity with proper intervals. She was quite sensible, and more composed; and had regained the power of putting out her tongue, which before she had lost. The pulse was at 106, and the tongue continued clean. Ordered to take at regular intervals a draught of infus. rosæ, made with decoct. cinchonæ, and to have occasionally a little Madeira wine.

" These favourable symptoms did not long continue. In the evening the pulse had got up to 120, and the heat had increased.

" From this time the patient became gradually weaker, her pulse was accelerated more and more, and her urine was again discharged involuntarily. She lived two days in a state of great anxiety and increasing restlessness, and died on Sunday night the 1st of July.

" This case appears to me an instance of a remarkable metastasis of of the Puerperal Fever; and had the disease been transferred to a less vital organ than the brain, a more happy crisis would probably have been the result. I have before mentioned that, at Aberdeen, the disease was not unfrequently transferred to the surface of the body, producing an erysipelas on the extremities, which proved a 'certain sign of salutary crisis.' And the transition of inflammatory affections of various kinds from one part of the body to another, is a fact well known in the practice of physic. In the case just related, it is observable, that, while the inflammation of the abdomen subsisted, the head was free from all complaint; and that, as soon as the inflammation was completely removed from the abdomen, to which it never in any degree returned, the head became affected with symptoms of inflammation, accompanied with evident marks of compression of the brain.*

---

" * *Metastasis propriè dicitur, quando, alio morbo quiescente, translata alio materia novum morbum excitat.*"

"Whatever other conclusions may be drawn from this case, the intire removal of the abdominal affection, and the appearance of the blood, which was of a firmer texture than any I had ever seen, both tended to confirm me in the propriety of bleeding in the disease under consideration." 91.

This case is of a *mixed* character. We are far from regarding it as one of pure puerperal peritonitis; we even doubt whether there was any peritoneal inflammation at all; but there can be no doubt but that there were intestinal inflammation, and some of the effects of loss of blood. The case began with shivering, succeeded by heat, and the attack was soon followed by an "astonishing quantity," and, the next day, by a "surprising quantity" of fæces, and by a "complete cessation" of the pain of the abdomen. On the third day an event occurred which is exceedingly common in cases of intestinal irritation, viz. an attack of affection of the head,—pain, with giddiness and ringing of the ears, the face being flushed and the pulse frequent; a somewhat similar seizure was repeated on the succeeding day, viz. "a sense of great confusion and noise," accompanied with much heat and flushing of the face, from mental excitement. Afterwards, there were indications of a paralytic affection; in a day or two more, restlessness and incessant talking; "in the course of this day the abdomen became tumid from flatus confined in the bowels; the tumefaction being unattended by pain or soreness, and entirely subsiding as soon as evacuations were procured by a glyster." The patient rallied the next day, became "quite sensible and more composed;" but, few patients recover from a "rattling in the breathing," a symptom which had been remarked the day before, and which is, if accurately observed in its very commencement, amongst the first, if not the very first of the symptoms of sinking from loss of blood.

We began our observations on this case, by observing that it was one of a *mixed* character. We doubt not that there was intestinal irritation,—we are certain that some of the symptoms arose from loss of blood; whether the affection of the abdomen was inflammation,—and what was the nature and the cause of the affection of the head, we will not pretend to determine; but we are far from being of the author's opinion of its being a case of metastasis; we cannot say all that we have thought on this point; but we think it right to observe, that such cases are frequently examples of the varied and successive effects of intestinal irritation. Admitting too that there was serious effusion or other morbid state within the head, this is, as we know, sometimes an effect of loss of blood; a most instructive case of this kind is published by the late Dr. Denman to which we beg to refer our readers.*

The tenth case, which is short, we transcribe for the sake of making one or two remarks.

"Mrs. S.———— was brought to bed on the 5th of July 1810, about nine o'clock in the morning. In her former labours she had been subject to a relaxation of the uterus after delivery, which usually occasioned a considerable flooding. Her discharge at this time was copious; but, being aware of the tendency to hæmorrhage, I was able, by suitable means, to keep it within moderate bounds.

"On the following day, at three o'clock in the afternoon, I was called to her in haste, on account of an excruciating pain which had suddenly seized the abdomen. It continued for half an hour without remission; but, before my arrival, it had ceased. As the pain was not preceded by rigor, and the pulse was not accelerated, I could not conclude the case to be one of Puerperal Fever; and therefore satisfied myself with prescribing an opening medicine, and requesting to be sent for immediately, if the pain should return.

"Having heard no more from the patient, I visited her late in the evening; and then found that the pain had returned, but with a less degree of severity; and, having had regular remissions, it had been mistaken for the common after-pain, and had therefore created little alarm. The abdomen had become very tender, and the pulse frequent.

"No doubt now remained on my mind of the nature of the disease; and though the attack was less distinctly marked, than in most of the cases which I had seen, my later experience warrants me in concluding, that the disease would soon have proved fatal, had not vigorous means been employed to check its progress. As night was approaching, I feared to wait till the symptoms became more urgent; and therefore, notwithstanding my reluctance to copious bleeding was not quite overcome, I immediately took from the arm a large basin full (about twenty ounces) of blood, and directed a continuation of the purgative. A cathartic clyster was also injected. The pain was diminished, while the blood was flowing, and on the following morning it was nearly gone; the fever had also greatly subsided. The bowels had been freely evacuated, yet I thought it advisable to maintain the purging undiminished for another day; and then it was suffered gradually to abate. The patient recovered without further complaint.

"Thus was an immediate stop put to the disease, which, had the bleeding been omitted, or deferred until morning, would, in all probability, have been irremediable. For though the first attack was, in some respects, less alarming than in many other cases; yet its early period, the severity of the pain, the consequent soreness of the abdomen, and the rapid increase of the pulse, clearly point it out as a genuine, and not a very

* Trans. of a Soc. for Imp. of Med. Knowl. v. 3, p. 315.

slight case of the prevailing epidemic. Perhaps the previous hæmorrhage might, in some degree, have obviated its violence." 94.

We agree with Mr. Hey that this was indeed a case of puerperal peritonitis; and it illustrates, in an instructive manner, an observation we have frequently made, that attack of that formidable malady is *not* necessarily ushered in by severe rigor, the pulse not necessarily very frequent, or the skin very hot, or the head affected. On the contrary, peritonitis is often insidious, detected in its early stages, on a careful examination only, and very apt to be overlooked during the first hours of its existence; in a short time, indeed, the pain and the state of the pulse sufficiently indicate the nature of the disease, and fix the attention of the practitioner; but the loss of that short time may cost the patient's life. We do not wish these remarks to be taken absolutely; in this as in other instances, "nulla perpetua præcepta;" there may be severe rigor,—there may be great heat of skin with inflammation; all we wish to observe is, that these symptoms are not constant; and we would just hint, that in many cases, they depend on a superadded cause, and that frequently intestinal irritation. Shivering in a slighter degree, and less followed by the other symptoms, is not unusual perhaps in pure peritonitis.

We have already noticed a case in which erysipelas occurred; in the eleventh case there was mortification of one of the extremities.

Nothing can be more instructive than the other cases given in Mr. Hey's work; the observations made by the author are all along extremely good. He remarks the absence of rigor in some decided cases of inflammation. The observations interspersed on the effects of blood-letting are excellent as applicable in cases of inflammation, and should be read again and again. There is little notice taken of affection of the head in all the cases which are plainly inflammatory. The skin sometimes becomes hot in the after periods of the disease, a circumstance which we have frequently noticed, and which we have sometimes ascribed to the remedies, and Mr. Hey frequently cautions against nursing, the attempt having often been immediately followed by an attack of the disease. We transcribe the twenty-first case, as exemplifying these remarks, and the usual character and remedies of true puerperal peritonitis.

"August 3rd 1812, at one o'clock in the morning, the wife of J. W—— of Hunslet, a woman of rather delicate appearance, was delivered by a midwife of her 12th child, after an easy labour of about an hour. Her discharge both at the time of labour and afterwards, was said to be copious, but not excessive. On the following morning she had a shivering fit, which was not, however, succeeded by pain; and she remained quite well throughout the day. The after-pains were slight.

"5th. At four o'clock in the morning, she was suddenly seized, without any previous chilliness, with a violent pain in the body resembling labour-pain, but of much longer duration. It increased progressively during the day; and in the intervals, which were not longer than a quarter of an hour, the abdomen was sore.

"I first saw her between four and five in the afternoon, and found her crying out in pain like a woman in labour. The remissions were now very short. There was little heat in the skin, and the countenance was pale; the tongue was clean and moist; the pulse about 112 and hard. The head was no way disordered. The abdomen was not swelled, nor the uterus distinctly to be felt. Pressure on the hypogastrium excited pain; but not in that great degree which is common in this complaint; and motion of the body was effected with tolerable ease. The child had sucked several times on the preceding day, but only once on this day; and that had greatly aggravated the pain. The breasts were now quite flaccid. The patient had taken some opening medicine, which had produced one loose evacuation in the morning.

"The symptoms, in this case, were not the most alarming, considering that thirteen hours had elapsed since the commencement of the disease; but the pain was violent; and the loss of time was more than a counterbalance to the apparent mildness of the other symptoms. I was therefore satisfied that large bleeding, in the first instance, was necessary; especially as night was approaching, and the patient lived at some distance from me. I first took away twenty-five ounces of blood, without producing any degree of faintness; when I closed the orifice for a few moments, till another basin was procured; and then drew nine ounces more. She was now disposed to faint, and the pain was much diminished. I put my finger on the orifice, and waited a while. The faintness soon went off, and the pain returned; I therefore took away six ounces more, making in the whole forty ounces. The patient becoming again very faint, I tied up the arm: she soon recovered, and remained easy. Pulse 88. A clyster was injected as soon as it could be prepared, which in ten minutes produced a very copious evacuation of solid fæces. At six *p. m.*, I gave a bolus with half a dram of jalap and four grains of calomel; and left directions that three table spoonfuls of the cathartic solution should be taken every two hours, till the bowels should be well opened, beginning two hours after taking the bolus.

"*Half-past ten, p. m.* The pain had returned soon after I left her, and with as much severity as before the bleeding. She had had three small watery stools, which did not appear to be the effect of the purgatives. The heat of the skin was now considerable, and was attended with much restlessness. The pulse was at 120, and still hard. The tongue was rather white, and the abdomen was much more tender; particularly in the region of the uterus, which had become enlarged, and easily distinguishable. This increase of all the symptoms since my former visit, seemed not only to justify the quantity of blood then taken; but to require a further evacuation. I tied up the arm, and took eight ounces from the same orifice; when, the patient growing faint, I desisted. The pain was much alleviated by this second bleeding, and the pulse came down to 84. I ordered the solution to be taken every hour.

"6th. *Eight, a. m.* She had remained nearly free from pain all the night; the soreness had greatly abated, the uterus was diminished in size, and she had slept several hours. The skin was moist and of a natural heat; the pulse at 100. She had taken above two ounces of magnes. sulphas, besides the purging bolus; and had had many small evacuations, which, however, contained but little fæces. Two boluses were therefore prescribed, with fifteen grains of jalap and two of calomel in each, to be taken with an interval of two hours; and the solution was ordered to be afterwards continued.

"*Six, p. m.* Both the boluses had been taken, and the remainder of the third ounce of magnes. sulphas, which had procured a great number of natural stools. The patient continued free from pain; the soreness of the abdomen was quite gone, and the uterus was scarcely to be felt.

"7th. She had slept the greatest part of the night, and the pulse was at 84. The bowels were kept open, and she continued convalescent." 135.

But we must hasten to a conclusion, and are therefore compelled to pass over the remaining cases which are given in this interesting work. They are we think more generally cases of inflammation than some which are given in the earlier part of the volume, and display in the most decided manner the efficacy of blood-letting in such cases. For many excellent remarks and for the rules for the use of this remedy we must refer to the treatise itself.

The appendix contains four cases on which we must, however, make a few observations. Like those in the beginning of the epidemic, these cases do not appear to be unmixed examples of peritonitis. Their author himself seems to be aware that they present some "variety," and especially that there was more affection of the *head* than he had observed at the commencement of the epidemic. In fact, not one of these four cases is pure peritonitis. The first is greatly, at least, intestinal irritation; the second presents many of the effects of loss of blood; the third forms an example of that affection, noticed in our arrangement of puerperal cases, as inflammation of the substance of the uterus or ovaria, frequently leading to suppuration; these cases have not been sufficiently attended to or accurately described,—they are not unfrequent, and certainly demand a modified mode of treatment, and in particular a stricter application of *local* remedies; the abscess sometimes points externally in some part of the lower region of the abdomen, and sometimes bursts into the rectum. The fourth and last case in the appendix, is one merely of intestinal irritation.

Although we have thus ventured to express our candid opinion, and although that opinion is frequently at variance with that of our author, we part with him with sentiments of sincere and unfeigned respect; we have greatly benefitted by the perusal of his excellent work, and are sure that all who read it, will do the same. And we offer our observations as hints merely, in aid of future inquiry, with the conviction that, if they be so received, they will lead to some practical and beneficial results.

Before we dismiss the subject, we wish shortly to recapitulate the scope of our observations. We believe then pure puerperal peritonitis to be frequently more insidious, less attended by rigor, heat, affection of the head, and whiteness of the tongue, than intestinal irritation: in the latter affection the attack, and the changes from better to worse, are frequently sudden and severe, marked by severe rigor, which is in its turn, followed by great heat, thirst, and affection of the head; in the former, the attack is sometimes insidious, there are fewer changes and repetitions of the attack, the course is more uniform, the rigor less severe, and sometimes altogether absent; the heat, thirst, whiteness of the tongue, and affection of the head, less, or totally wanting. It is observable too that these states occur *together*, or are nearly or totally absent, in different instances. An examination of Mr. Hey's cases will illustrate and confirm this remark.

The result of our observations then is this:—If there be no rigor, or only slight rigor, still *suspect* inflammation; if the surface is cool, the head unaffected,—if the tongue be clean or the pulse, even, little changed, *still suspect peritonitis*, and carefully examine the abdomen. If, on the contrary, there be severe rigor, if the heat and thirst be great, if the tongue be loaded, and if the head be affected,—whatever other affection there may be,—turn your attention to the state of the intestinal contents, and suspect irritation from this source. We do not counsel you to neglect the lancet if there be symptoms of inflammation; it may be a *mixed* case; therefore do that, but leave not this undone.

In particular, let us seize every opportunity of pursuing our researches into the morbid anatomy of puerperal diseases, and we shall come at last to understand that remark quoted by Dr. Denman, that "in the dissections of some who have died of this disease, (puerperal fever,) no appearances of inflammation have been discovered."

